# A Rare Case of Leiomyoma‐Associated Cutaneous Sinus Tract Secondary to an Infected Uterine Leiomyoma Without Prior Myomectomy

**DOI:** 10.1155/crog/3211851

**Published:** 2026-06-10

**Authors:** Brahmana Askandar Tjokroprawiro, Khoirunnisa Novitasari, Renata Alya Ulhaq, Hanif Ardiansyah Sulistya, Teuku Mirza Iskandar

**Affiliations:** ^1^ Department of Obstetrics and Gynecology, Faculty of Medicine, Universitas Airlangga, Surabaya, Indonesia, unair.ac.id; ^2^ Department of Obstetrics and Gynecology, Dr. Soetomo General Academic Hospital, Surabaya, Indonesia, rsudrsoetomo.jatimprov.go.id; ^3^ Gynecologic-Oncology Division of Obstetrics and Gynecology, Faculty of Medicine, Universitas Diponegoro/Dr. Kariadi General Hospital, Semarang, Central Java, Indonesia

**Keywords:** giant leiomyoma, infected, uterocutaneous fistula

## Abstract

**Background:**

Leiomyoma‐associated cutaneous sinus tracts are exceptionally rare and typically associated with prior uterine surgeries, such as cesarean section or myomectomy. Fistula formation from an infected uterine leiomyoma involving the uterine parenchyma, without prior myomectomy, represents an uncommon clinical presentation.

**Case:**

A 22‐year‐old woman with a history of cesarean section 5 months prior presented with purulent discharge from an abdominal mass. Imaging revealed a large uterine mass containing gas, with a fistulous tract extending to the abdominal skin. Laparotomy confirmed a fistulous tract extending from the abdominal skin to an infected leiomyoma within the uterine fundal parenchyma, without demonstrable communication with the endometrial cavity. Surgical intervention consisted of fistula closure, myomectomy, and drainage. Histopathological examination confirmed the presence of a uterine leiomyoma with chronic suppurative inflammation, consistent with an infected myoma. The patient remained asymptomatic for 15 months following surgery.

**Conclusion:**

This case highlights a rare variant of leiomyoma‐associated cutaneous sinus tract arising from an infected uterine leiomyoma involving the uterine parenchyma. Careful diagnostic evaluation and tailored surgical management resulted in complete recovery and a favorable long‐term outcome.

## 1. Introduction

Leiomyoma‐associated cutaneous sinus tract is a rare condition, primarily resulting from surgical complications, such as multiple cesarean sections, incomplete hysterorrhaphy, and postoperative infections [1]. Over the past two decades, fewer than 15 cases have been reported worldwide, highlighting its rarity [2]. Owing to its rarity, the clinical presentation may vary, often complicating the diagnosis of leiomyoma‐associated cutaneous sinus tract. Effective management requires a comprehensive understanding of disease etiology and potential consequences; recent case reports emphasize the need for appropriate diagnosis, tailored therapeutic methods, and increased awareness to improve outcomes. Although leiomyomas are the most prevalent gynecologic pathological findings, their association with the formation of a leiomyoma‐associated cutaneous sinus tract is extremely rare and has been documented only following surgical interventions such as cesarean delivery or myomectomy [3, 4]. This case report highlights the occurrence of a leiomyoma‐associated cutaneous sinus tract secondary to an infected giant uterine leiomyoma.

## 2. Case Report

A 22‐year‐old woman presented to our hospital with the primary complaint of a small abdominal bulge at the site of her previous surgical scar that had persisted for 1 month. The mass was accompanied by mild lower abdominal discomfort and intermittent discharge of a clear, whitish‐yellow fluid. The patient denied fever or chills, reported regular menstrual cycles, and had one living child. Five months prior, she had undergone a cesarean section at a different private hospital due to intrauterine fetal death (IUFD) at 6 months of gestation and a history of previous cesarean delivery.

Approximately 1 month post‐surgery, she observed the emergence of a small lump on the abdominal skin at the site of her previous surgical scar, which intermittently released purulent fluid. Initially, she was empirically treated with antibiotics without prior culture and sensitivity testing and was subsequently discharged. However, 2 months later, she underwent resuturing of the wound due to persistent discharge, which temporarily reduced the pus output but was followed by a recurrence of both purulent and serous fluid. In retrospect, targeted antibiotic therapy guided by culture and sensitivity results may have led to earlier resolution of the infection. As symptoms persisted, further evaluations were conducted at our facility.

Upon examination, a small, soft tumor exuding a whitish‐yellow discharge was observed at the previous surgical site, possibly linked to the abdominal cavity. The uterine fundus was palpated 3 cm above the umbilicus. Laboratory findings revealed mild anemia with a hemoglobin level of 10.8 g/dL (reference range: 11.0–14.7 g/dL), leukocytosis with a white blood cell count of 13.45 × 10^3^/*μ*L (reference range: 3.37–10.0 × 10^3^/*μ*L), and thrombocytosis with a platelet count of 490 × 10^3^/*μ*L (reference range: 150–450 × 10^3^/*μ*L). Serum albumin levels were within normal limits at 3.43 g/dL (reference range: 3.4–5.0 g/dL)

Abdominal ultrasonography revealed an enlarged uterus with a gas‐containing lesion and internal echoes within the fundal parenchyma, measuring 11.3 × 10 × 13 cm and with well‐defined margins. Pelvic magnetic resonance imaging (MRI) with contrast demonstrated a 14 × 13 × 9 cm abscess in the uterine fundal parenchyma, with strong margins and contrast enhancement. Additionally, a 3.1‐cm uterocutaneous fistula was identified (Figure 1).

**Figure 1:**
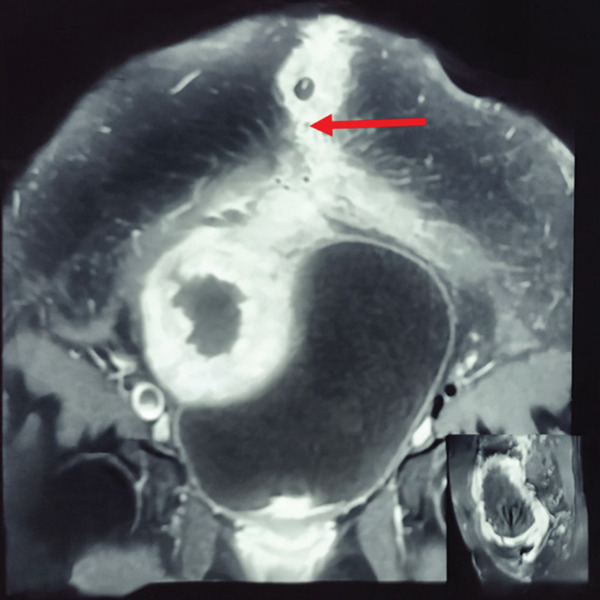
Preoperative pelvic magnetic resonance imaging demonstrating a large fundal uterine mass (arrowhead) with internal gas and abscess formation, and a fistulous tract extending toward the anterior abdominal wall.

The patient subsequently underwent an exploratory laparotomy via a midline incision extending 3 cm above the umbilicus to the symphysis pubis. Intraoperative findings revealed a fistulous tract extending from the abdominal skin to the uterus, originating from a giant infected myoma with an abscess formation on the anterior uterine wall. Surgical procedures included drainage, fistulectomy, myomectomy, and repair of the anterior uterine fundal wall defect resulting from myoma excision (Figure 2).

**Figure 2:**
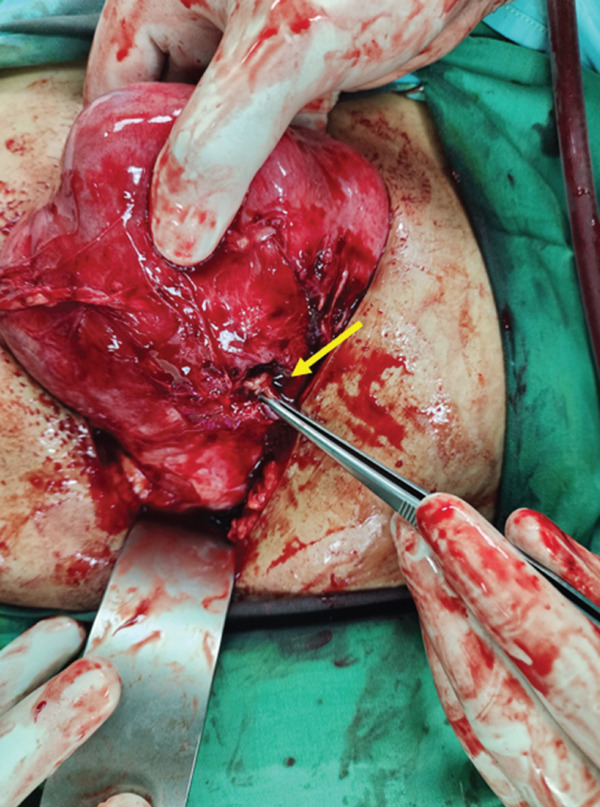
Intraoperative findings showing a fistulous tract extending from the abdominal skin to an infected uterine leiomyoma located within the uterine fundal parenchyma. The arrowhead indicates the tract′s origin from the infected myoma; notably, the tract is distinct from and not associated with the previous cesarean section incision site.

Gross examination of the excised mass revealed a poorly demarcated lesion measuring approximately 11 × 5 × 5 cm with a white‐gray to yellow appearance and a solid, spongy consistency, containing a 5‐cm perforated cavity. Histopathological analysis confirmed the diagnosis of a uterine leiomyoma with chronic suppurative inflammation, consistent with abscess formation within the myoma (Figure 3).

**Figure 3:**
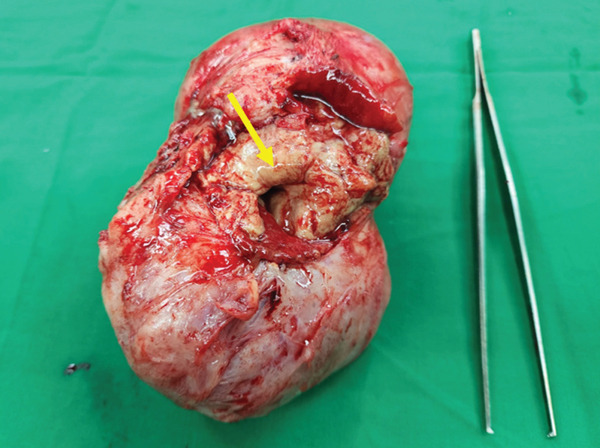
Giant uterine myoma with abscess formation (arrowhead).

Microbiological cultures of the surgical pus identified *Serratia marcescens* as the causative organism. Antibiotic susceptibility testing revealed sensitivity to cefoperazone‐sulbactam, gentamicin, ciprofloxacin, and levofloxacin. The patient was treated with cefoperazone‐sulbactam, resulting in marked clinical improvement.

The postoperative course was uneventful. The patient returned for follow‐up in good condition, with complete wound healing observed within 3 weeks. Fifteen months after surgery, the patient remained asymptomatic, with no recurrence or complications.

## 3. Discussion

Surgical intervention is the most significant risk factor for uterocutaneous fistula formation, likely due to the disruption of uterine wall integrity and subsequent adhesion formation. A uterocutaneous fistula is classically defined as an abnormal tract connecting the endometrial cavity to the abdominal skin; however, atypical presentations, with fistulous tracts arising from the uterine wall or parenchyma without clear endometrial cavity communication, have also been reported. It is acknowledged that because the tract in this case did not communicate with the endometrial cavity, the term “leiomyoma‐associated sinus tract” may be more precise by definition. Nevertheless, the term “uterocutaneous fistula” is retained here to maintain consistency with the broader literature describing similar atypical presentations. This condition has been reported following various uterine procedures, including cesarean section, myomectomy [3–5], and uterine artery embolization (UAE), a treatment option for leiomyomas. UAE induces ischemia and necrosis in uterine leiomyomas, leading to degenerative changes and inflammatory responses in the surrounding tissue [6, 7]. Although most cases are associated with prior surgical procedures, a leiomyoma‐associated cutaneous sinus tract can develop spontaneously in the presence of benign uterine leiomyomas, even without a history of surgery, as reported by Anderson in 2020 [8].

In this case, the fistula formation may have resulted from the large size of the leiomyoma and its protrusion toward the peritoneal or subserosal surface. The resulting mechanical pressure and local ischemia could have caused necrosis of the surrounding tissue, leading to progressive tissue breakdown and eventual tract formation [9, 10]. Areas of necrosis may develop into abscesses that gradually erode the surrounding tissues, ultimately forming a tract connecting the uterus to the skin surface. In some cases, a giant leiomyoma may undergo degenerative changes such as cystic or red degeneration, which weaken the structural integrity of the myoma and adjacent tissues. This can trigger a localized inflammatory response, increasing the susceptibility of the tissues to fistula formation [3]. This case represents a rare presentation of *leiomyoma-associated cutaneous sinus tract*, in which the fistulous tract arose from an infected uterine leiomyoma involving the uterine parenchyma rather than from the uterine incision site. This atypical presentation poses challenges in both diagnosis and management. Additionally, although a temporal relationship with the prior cesarean section cannot be entirely excluded, intraoperative findings suggested that infection and abscess formation within the leiomyoma were central in the development of the sinus tract.

Uterocutaneous fistulas typically present with purulent discharge from the abdominal wall. Ultrasonography can help identify the fistulous tract and its association with underlying myomas, whereas MRI provides more detailed visualization of the anatomical structures and surrounding tissues [10]. Treatment should be tailored to the patient′s clinical condition, including the underlying cause, overall health status, and fertility desire. While gonadotropin‐releasing hormone (GnRH) agonists have been reported as a conservative approach for uterocutaneous fistula with endometrial communication or hormonal contribution, their role is limited when the primary etiology is infectious, as in the present case. In infection‐driven presentations, adequate source control through surgical drainage, antimicrobial therapy guided by culture and sensitivity results, and excision of the infected focus remain the cornerstone of management [9, 11, 12]. However, surgical intervention, which typically involves total hysterectomy or complete excision of the fistulous tract followed by uterine repair, remains the standard of care in most cases owing to the complexity of the condition. In this patient, sinus tract excision, myomectomy, and uterine repair were performed, followed by a 5‐day course of targeted antibiotic therapy (cefoperazone–sulbactam) given the presence of an infected myoma, her young age, and the desire to preserve fertility. The patient returned for follow‐up in good condition, with complete wound healing within 3 weeks, and has remained asymptomatic to date.

## 4. Conclusions

This case contributes to the limited number of reported cases of *leiomyoma-associated cutaneous sinus tracts* caused by an infected uterine leiomyoma without prior myomectomy. It highlights the importance of understanding the underlying pathophysiology, maintaining a high index of suspicion for atypical presentations, and implementing individualized management strategies. Early diagnosis and tailored surgical intervention led to a complete recovery and excellent long‐term outcomes.

## Funding

No funding was received for this manuscript.

## Disclosure

All authors have read and approved the final version of the manuscript. The corresponding author had full access to all data in this study and takes full responsibility for the integrity and accuracy of the data analysis. This case report complies with the CARE Consensus. The corresponding author, Brahmana Askandar Tjokroprawiro, affirms that this manuscript is an honest, accurate, and transparent account of the study; that no important aspects of the study have been omitted; and that any discrepancies from the study as planned have been explained.

## Ethics Statement

The authors have nothing to report.

## Consent

Written informed consent was obtained from the subject and/or guardian.

## Conflicts of Interest

The authors declare no conflicts of interest.

## Data Availability

The data that support the findings of this study are available on request from the corresponding author. The data are not publicly available due to privacy or ethical restrictions.
